# Ambulatory isolated diastolic hypertension and risk of left ventricular hypertrophy in children with primary and secondary hypertension

**DOI:** 10.1007/s00467-024-06457-8

**Published:** 2024-07-18

**Authors:** Robert L. Myette, Łukasz Obrycki, Mieczysław Litwin, Tomáš Seeman, Terezie Šuláková, Janusz Feber

**Affiliations:** 1 Division of Nephrology, Department of Pediatrics, Children’s Hospital of Eastern Ontario, University of Ottawa, Ottawa, Canada; 2 Kidney Research Center, Department of Cellular and Molecular Medicine, The Ottawa Hospital Research Institute, University of Ottawa, Ottawa, Canada; 3 Department of Nephrology, Kidney Transplantation and Hypertension, Children’s Memorial Health Institute, Warsaw, Poland; 4 Department of Pediatrics, Charles University Prague, 2nd Faculty of Medicine, Prague, Czech Republic; 5 Department of Pediatrics, University Hospital Ostrava, Ostrava, Czech Republic; 6 Medical Faculty, University of Ostrava, Ostrava, Czech Republic

**Keywords:** Hypertension, Children, Diastolic blood pressure, Ambulatory blood pressure monitoring

## Abstract

**Background:**

Pediatric blood pressure (BP) assessment and management is increasingly important. Uncontrolled systolic and combined hypertension leads to hypertension-mediated organ damage. The impact of isolated diastolic hypertension is less clearly understood.

**Methods:**

We analyzed the prevalence of ambulatory isolated diastolic hypertension (IDH) in primary (PH) and secondary (SH) hypertension, and associations with BMI *Z*-score (BMIz) and left ventricular mass index adjusted to the 95th percentile (aLVMI) in a large, multicenter cohort of hypertensive children. Hypertensive children were divided and analyzed in three ambulatory hypertension subgroups: 24-h, daytime, and nighttime. Specifically, we sought to determine the prevalence of ambulatory 24-h, daytime, or nighttime IDH.

**Results:**

Prevalence of IDH varied based on ambulatory phenotypes, ranging from 6 to 12%, and was highest in children with SH. Children with IDH tended to be more likely female and, in some cases, were leaner than those with isolated systolic hypertension (ISH). Despite previous pediatric studies suggesting no strong association between diastolic blood pressure and left ventricular hypertrophy (LVH), we observed that children with IDH were equally likely to have LVH and had comparable aLVMI to those with ISH and combined systolic-diastolic hypertension.

**Conclusions:**

In summary, ambulatory IDH appears to be a unique phenotype with a female sex, and younger age predilection, but equal risk for LVH in children with either PH or SH.

**Graphical abstract:**

**Figure:**
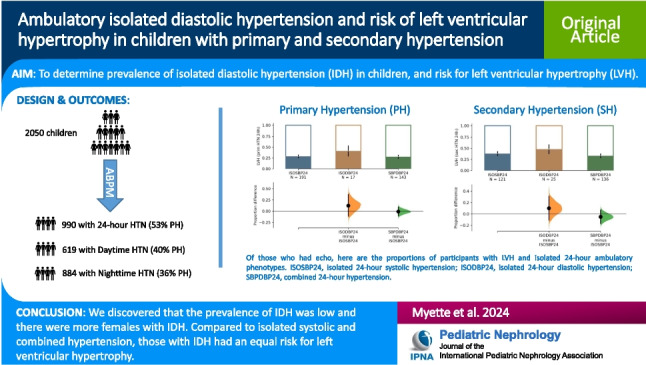
A higher-resolution version of the Graphical abstract is available as  Supplementary information

**Supplementary Information:**

The online version contains supplementary material available at 10.1007/s00467-024-06457-8.

## Introduction

Pediatric blood pressure (BP) assessment and management is increasingly important, given that 3–5% of children have hypertension, with an even higher number in obese children [1, 2]. It is well-defined that hypertension can lead to hypertension-mediated organ damage (HMOD), specifically mainly left ventricular hypertrophy (LVH). Recent studies have assessed the impact of office systolic hypertension [3], and office pre-hypertension [4], on LVH. The impact of ambulatory isolated diastolic hypertension (IDH) on HMOD is poorly understood.

Recently, it has been reported that subjects with IDH had a distinct clinical phenotype when compared to children with systolic-diastolic hypertension (SDH) or isolated systolic hypertension (ISH). Using National Health and Nutrition Examination Survey (NHANES) data, the authors discovered that generally healthy children with increased office DBP were younger, had a leaner body habitus, and were more likely female than those with ISH [5]. They also had a higher resting heart rate, and the authors concluded this might speak to a unique underlying pathophysiology associated with elevated DBP [5]. Importantly, the authors did not comment on differences observed between those with a primary hypertension (PH) or secondary hypertension (SH) diagnosis nor did they have ambulatory blood pressure (ABP) data.

Rasała et al. reviewed differences in anthropometric and biochemical parameters between children with PH and SH and observed children with SH were more likely to have higher DBP values on 24-h and nighttime monitoring, as well as higher DBP loads [6]; however, the authors did not evaluate echocardiography in relation to these findings.

It appears that distinct BP phenotypes may be associated with different etiologies and an unclear risk for HMOD. Therefore, our main objective was to analyze ABP phenotypes to determine a more accurate prevalence of ambulatory IDH in a large, international cohort of children with PH and SH, and to better characterize the risk of LVH related to ambulatory IDH.

## Methods

### Population

We retrospectively analyzed data from all consecutively enrolled children aged ≤ 18 years from four academic centers in three countries who were diagnosed with PH or SH from February 2012 to December 2021 and who underwent 24-h ambulatory blood pressure monitoring (ABPM). In total, there were 2050 patients recruited. Patients were recruited from The Children’s Hospital of Eastern Ontario, Ottawa, Ontario, Canada; The Children’s Memorial Health Institute, Warsaw, Poland; The Department of Pediatrics, Charles University Prague, 2nd Faculty of Medicine, Prague, Czech Republic; and The Department of Pediatrics, University Hospital Ostrava, Ostrava, Czech Republic. Ethics approval from each institution’s Research Ethics Board was obtained. Informed consent was obtained where required; however, certain institutions waived the need for informed consent due to the retrospective nature of the study.

### BP measurements

The 24-h ABPM was performed using a standard oscillometric ABPM device (Spacelabs Monitory 90207 or 90217). The appropriately sized cuff was applied to the non-dominant arm. Recordings were taken every 15–30 min during the day and every 30–60 min overnight. Recordings which lasted > 20 h or included at least 80% viable readings were considered valid and included in the study. For this study, we defined daytime BP as those values recorded between 08:00 and 20:00, while nighttime recordings were defined as those recordings between 00:00 and 06:00 [7]. In children < 13 years of age, the absolute summary values were converted into *Z*-scores according to ABPM normative data [7].

### Definition of ambulatory hypertension

We classified ambulatory hypertension, as well as normotension, using the most current AHA Scientific Statement from 2022 [8]. A *Z*-score of 1.65 represents the 95th percentile. Ambulatory normotension was defined as 24-h SBP and DBP *Z*-scores < 1.65 if < 13 years old, or 24-h BP < 125/75 and daytime BP < 130/80 and nighttime BP < 110/65 if $\ge$ 13 years old. Ambulatory hypertension definitions were as follows: for ISH or IDH, the *Z*-score values for SBP or DBP were $\ge$ 1.65 if the age was < 13 years; however, for those $\ge$ 13 years of age, ambulatory ISH was defined as SBP $\ge$ 125 mmHg (24-h period), SBP $\ge$ 130 mmHg (daytime period), or SBP $\ge$ 110 mmHg (nighttime period), with corresponding normotensive DBP values. Ambulatory IDH in those $\ge$ 13 years was DBP $\ge$ 75 mmHg (24-h period), DBP $\ge$ 80 mmHg (daytime period), or DBP $\ge$ 65 mmHg (nighttime period), with corresponding normotensive SBP values. Ambulatory combined systolic-diastolic hypertension (SDH) was a combination of the above ambulatory ISH and IDH.

### Primary and secondary hypertension definitions

The patients were recruited at respective centers, identified above. The attending physician who enrolled the patient into the study was responsible for determining the type of hypertension based on clinical expertise and confirming the diagnosis of hypertension using appropriate guidelines (European Society of Hypertension guidelines for European centers, and The American Academy of Pediatrics guidelines for Canadian centers). The SH group was comprised of a variety of diagnoses, including glomerular diseases, congenital anomalies of kidney and urinary tract, and endocrinological disorders. Main comparisons were made between those patients with PH and SH, categorized into different isolated ABP phenotypes.

### Echocardiography

Echocardiography was performed by skilled operators at the respective institutions. All measurements were performed and recorded in accordance with the American Society of Echocardiography guidelines [9]. LVM (Devereux Equation [10]) was converted to LVM index (LVMi) in units g/m^2.7^ and then adjusted to the 95th percentile (aLVMI) [11, 12]. LVH was defined as aLVMI > 1.0. Approximately 90% of children had echocardiography within 6 months of their ABPM.

### Classification of patients

ABP phenotypes (i.e., 24 h, daytime, nighttime) were analyzed independently. However, without office measurements, we are unable to provide data on white coat hypertension, masked hypertension, or true/sustained hypertension in this analysis. Patients classified as normotensive on ambulatory measurements were removed from the analysis. Based on definitions, there is some overlap in those with 24-h hypertension and those with either daytime or nighttime. Of the 2050 enrolled, after removal of normotensive children, there were 990 with 24-h hypertension, 619 with daytime hypertension, and 884 with nighttime hypertension. There was incomplete documentation of exposure to antihypertensives; however, of the patients with secondary hypertension, we have antihypertensive data on 234 of them, and 88% were on monotherapy.

### Statistical analysis

Categorical variables were expressed as *N* (counts) with percentages and compared using the Chi-squared test. Continuous variables were expressed as mean + / − standard deviation (SD) for normally distributed data, or median with interquartile range (IQR). Normality was assessed using the Shapiro–Wilk test. If one comparator was non-normally distributed, both comparators were taken as non-normally distributed and analyzed as such. Differences among office and ambulatory BP groups were evaluated using analysis of variance (ANOVA), or non-parametrically using Kruskal–Wallis testing. For post hoc pairwise comparisons, Tukey HSD was used for post hoc analysis when normally distributed, or Conover if non-normally distributed. Evaluation statistics with mean difference and 95% confidence intervals of the difference was used to analyze and visualize differences between blood pressure subgroups in aLVMi and proportions of sex and LVH. A *P* value of < 0.05 was considered statistically significant. All tests were performed using statistical software R (version 1.1.456) and Python (version 3.8.3) [13].

## Results

### Hypertensive children

Of the 990 children with 24-h hypertension, there were 524 (53%) with PH and 466 (47%) with SH (Table 1, A). Of the 619 children with daytime hypertension, there were 248 (40%) with PH and 371 (60%) with SH, summarized in Table 1, B. Of the 884 children with nighttime hypertension, there were 321 (36%) with PH and 563 (64%) with SH, summarized in Table 1, C.

**Table 1:** Anthropometric, biochemical, and blood pressure data for the overall cohort stratified by ABPM classification. 24-h (A); daytime (B); nighttime (C). Mean (SD); median [IQR]; *n* (%). BMI, body mass index; GFR, glomerular filtration rate; SBP, systolic blood pressure; DBP, diastolic blood pressure; LVMI, left ventricular mass index; aLVMI, adjusted LVMI; LVH, left ventricular hypertrophy

A	Primary	Secondary
24-h	Overall	Overall
*N* = 990	524 (53%)	466 (47%)
Age (years)	15.91 [13.77, 16.94]	15.00 [11.99, 16.76]
Female sex, *n* (%)	148 (28.2)	202 (43.3)
BMI (kg/m^2^)	24.10 [21.49, 28.73]	21.00 [17.57, 24.80]
BMI *Z*-score	1.32 [0.39, 2.26]	0.56 [− 0.29, 1.69]
BMI > 95%ile (*n*, %)	179 (41.0)	98 (25.7)
GFR (ml/min/1.73 m^2^)	108.11 (21.74)	93.34 (34.36)
24-h SBP	132.00 [127.00, 137.00]	129.40 [125.00, 135.00]
24-h SBP *Z*-score	2.04 [1.45, 2.65]	2.13 [1.49, 2.90]
24-h DBP	74.00 [69.00, 78.00]	75.00 [70.00, 81.00]
24-h DBP *Z*-score	1.08 [0.35, 1.88]	1.47 [0.61, 2.50]
LVMI	36.06 [32.23, 41.21]	36.63 [31.79, 43.84]
aLVMI	0.90 [0.79, 1.01]	0.94 [0.78, 1.08]
LVH (*n*, %)	102 (29.1)	103 (36.5)
B	Primary	Secondary
Daytime	Overall	Overall
*N* = 619	248 (40%)	371 (60%)
Age (years)	15.50 [13.21, 16.87]	14.97 [12.04, 16.78]
Female sex, *n* (%)	82 (33.1)	154 (41.5)
BMI (kg/m^2^)	24.15 [20.41, 29.58]	20.92 [17.50, 24.67]
BMI *Z*-score	1.39 [0.29, 2.44]	0.55 [− 0.41, 1.62]
BMI > 95%ile (*n*, %)	83 (45.6)	76 (24.3)
GFR (ml/min/1.73 m^2^)	108.14 (21.66)	92.59 (34.72)
Daytime SBP	135.00 [131.00, 140.00]	134.00 [130.00, 139.00]
Daytime SBP *Z*-score	1.84 [1.30, 2.32]	1.89 [1.35, 2.54]
Daytime DBP	76.00 [71.00, 82.25]	80.00 [73.00, 85.00]
Daytime DBP *Z*-score	0.64 [-0.20, 1.66]	1.21 [0.11, 2.28]
LVMI	35.65 [32.23, 41.25]	36.54 [31.36, 43.69]
aLVMI	0.91 [0.78, 1.07]	0.93 [0.78, 1.08]
LVH (*n*, %)	40 (33.6)	89 (36.9)
C	Primary	Secondary
Nighttime	Overall	Overall
*N* = 884	321 (36%)	563 (64%)
Age (years)	15.18 [13.13, 16.67]	15.24 [12.67, 16.73]
Female sex, n (%)	117 (36.4)	219 (38.9)
BMI (kg/m^2^)	24.10 [20.15, 29.99]	21.76 [18.03, 25.60]
BMI *Z*-score	1.46 [0.20, 2.57]	0.73 [− 0.22, 1.83]
BMI > 95%ile (*n*, %)	111 (47.8)	133 (27.9)
GFR (ml/min/1.73 m^2^)	107.49 (20.91)	91.97 (35.81)
Nighttime SBP	119.00 [114.00, 125.00]	118.00 [112.00, 124.00]
Nighttime SBP *Z*-score	1.88 [1.25, 2.58]	1.76 [1.10, 2.56]
Nighttime DBP	65.00 [59.00, 69.00]	66.00 [60.00, 71.00]
Nighttime DBP *Z*-score	1.43 [0.38, 2.12]	1.62 [0.60, 2.50]
LVMI	35.43 [32.18, 41.36]	36.53 [31.40, 42.81]
aLVMI	0.89 [0.77, 1.03]	0.91 [0.78, 1.05]
LVH (*n*, %)	41 (29.7)	114 (33.3)

### Children with 24-h hypertension

**Fig. 1:**
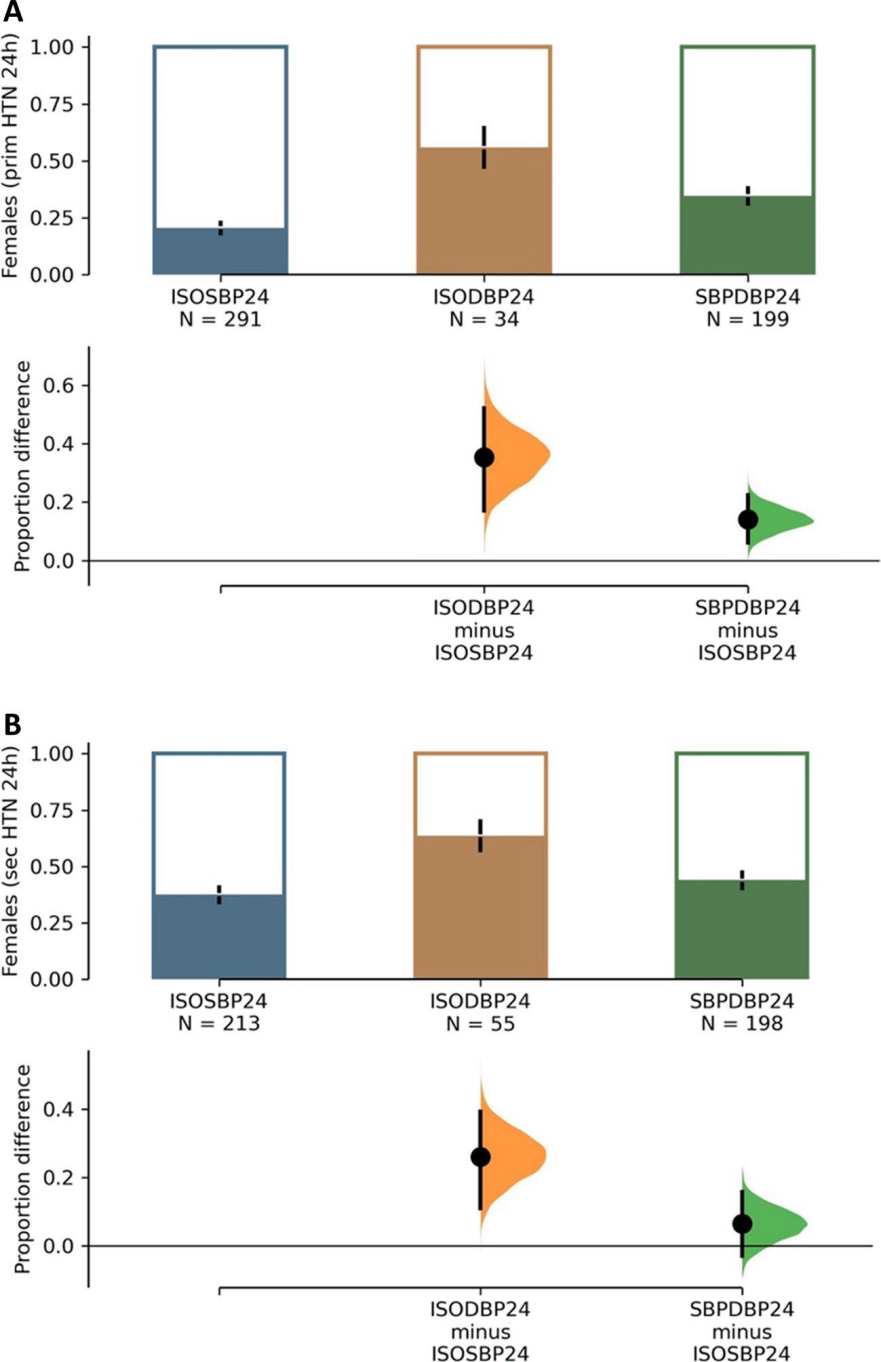
**A** Evaluation statistics with mean difference and 95% confidence intervals of the difference shown for females with primary hypertension (PH) across the ambulatory phenotypes. Isolated diastolic hypertension was higher in females with PH and 24-h hypertension. ISOSBP24, isolated 24-h systolic hypertension; ISODBP24, isolated 24-h diastolic hypertension; SBPDBP24, combined 24-h hypertension. **B** Evaluation statistics with mean difference and 95% confidence intervals of the difference shown for females with secondary hypertension (SH) across the ambulatory phenotypes. Isolated diastolic hypertension was higher in females with SH and 24-h hypertension. ISOSBP24, isolated 24-h systolic hypertension; ISODBP24, isolated 24-h diastolic hypertension; SBPDBP24, combined 24-h hypertension

**Fig. 2:**
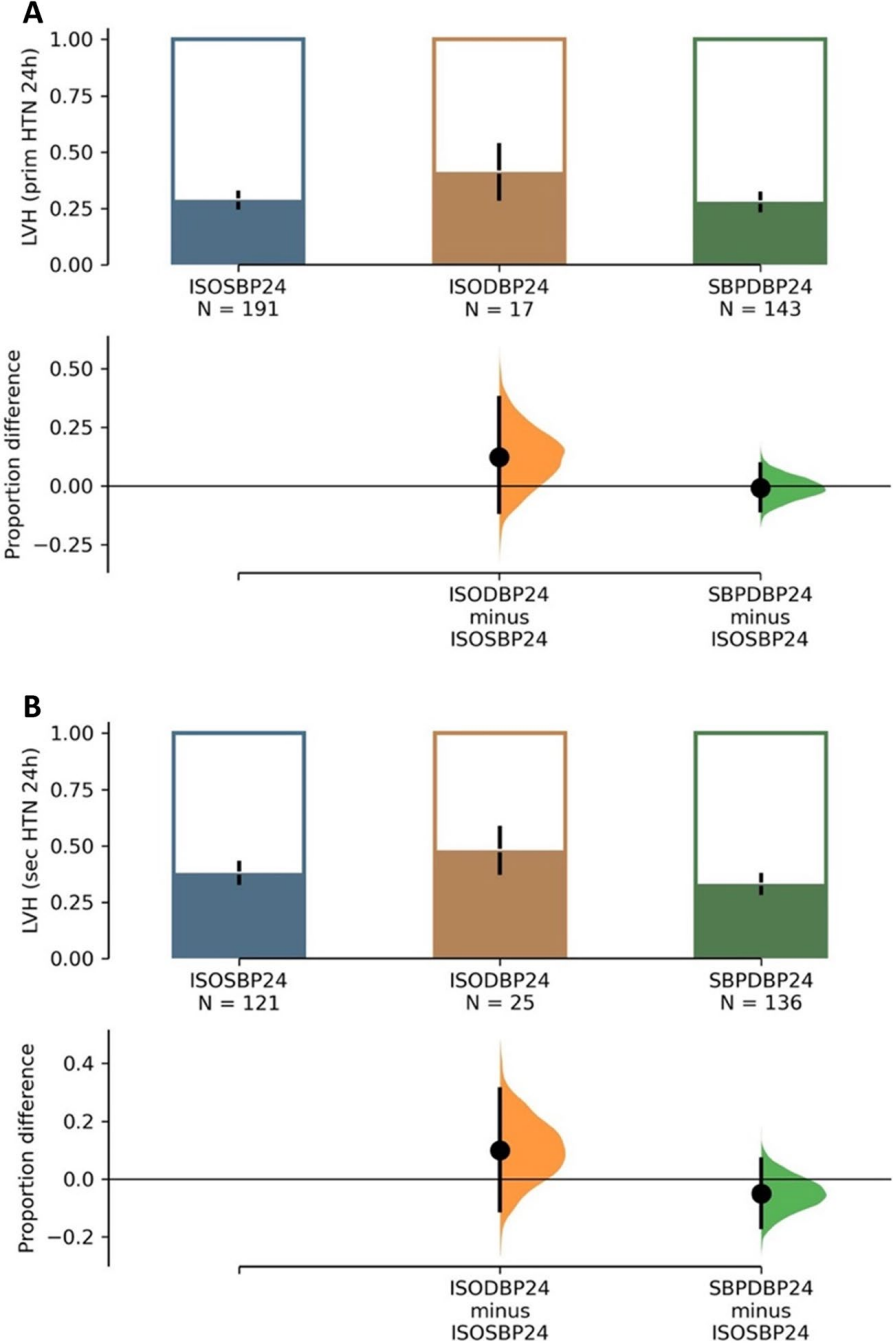
**A** Evaluation statistics with mean difference and 95% confidence intervals of the difference shown for LVH prevalence in those with primary hypertension (PH) and 24-h hypertension. This reveals that children with PH and 24-h IDH had non-significantly different, and possibly higher, chance of LVH. ISOSBP24, isolated 24-h systolic hypertension; ISODBP24, isolated 24-h diastolic hypertension; SBPDBP24, combined 24-h hypertension. **B** Evaluation statistics with mean difference and 95% confidence intervals of the difference shown for LVH prevalence in those with secondary hypertension (SH) and 24-h hypertension. This reveals that children with SH and 24-h IDH had non-significantly different, and possibly higher, chance of LVH. ISOSBP24, isolated 24-h systolic hypertension; ISODBP24, isolated 24-h diastolic hypertension; SBPDBP24, combined 24-h hypertension

Children with IDH were of similar age compared to children with ISH and SDH, except for PH children with SDH, whose age was significantly higher compared to ISH (mean difference = 0.87, 95% confidence interval (95% CI) = 0.42–1.32, *P* = 0.0003; Table 2). The proportion of females was significantly higher in IDH in both PH (Fig. 1A) and SH patients (Fig. 1B). The mean difference in the proportion of females (95% CI) from the ISH group was 35% (17–52%; *P* = 0.001) in PH and 26% (11–39%; *P* = 0.001) in SH. There were no significant differences in BMI *Z*-scores and proportions of obese children (BMI > 95th percentile) among ABP phenotypes, except for children with SH and SDH who had lower BMI *Z*-scores and lower proportion of BMI > 95th percentile. Interestingly, children with IDH had similar aLVMi as children with ISH and SDH in PH and SH (Table 2). There were also no significant differences in LVH between 24-h ABP phenotypes (Fig. 2A, B). Children with IDH had the highest numerical proportion of LVH but without statistical significance. We observed expected differences in absolute BP values and BP *Z*-scores across ABP groups for both PH and SH, summarized in Supplementary Table S1A.

**Table 2:** Anthropometric, biochemical, and blood pressure data for the overall cohort stratified by 24-h ABPM classification. Mean (SD); median [IQR]; *n* (%). BMI, body mass index; LVMI, left ventricular mass index; aLVMI, adjusted LVMI; LVH, left ventricular hypertrophy. Not all participants had echocardiogram, denominator for LVH (%) calculation included. *P* value column, ANOVA across three groups. * = significantly different from ISH, post hoc (*P* < 0.05). ISH used as the primary comparator due to highest number of patients. IDH highlighted in grey

24-h	Primary			*P*	Secondary			*P*
	24-h ISH	24-h IDH	24-h SDH		24-h ISH	24-h IDH	24-h SDH	
*N* (%)	291 (55.5%)	34 (6.5%)	199 (38%)		213 (45.7%)	55 (11.8%)	198 (42.5%)	
Age (years)	15.49 [13.49, 16.83]	15.97 [13.51, 16.85]	16.21 [14.69, 16.99]*	0.004	14.97 [12.02, 16.66]	14.76 [11.88, 17.08]	15.11 [11.95, 16.81]	0.922
Female sex, *n* (%)	60 (20.6)	19 (55.9) *	69 (34.7) *	< 0.001	80 (37.6)	35 (63.6) *	87 (43.9)	0.002
BMI (kg/m^2^)	23.67 [21.42, 28.72]	22.85 [20.01, 29.58]	24.63 [22.10, 28.56]	0.471	21.74 [19.24, 25.95]	21.69 [18.12, 26.68]	19.71 [17.09, 23.56]*	0.001
BMI *Z*-score	1.35 [0.38, 2.31]	1.44 [0.50, 2.25]	1.28 [0.39, 2.20]	0.91	0.91 [− 0.04, 2.00]	0.56 [− 0.24, 1.77]	0.30 [− 0.70, 1.26]*	0.001
BMI > 95%ile (*n*, %)	101 (41.1)	11 (40.7)	67 (40.9)	0.999	50 (30.7)	15 (31.2)	33 (19.4) *	0.041
LVMI	35.93 [32.61, 41.03]	38.13 [31.07, 40.43]	36.13 [32.04, 41.34]	0.874	37.15 [33.28, 43.02]	40.00 [29.73, 45.34]	35.61 [30.78, 42.38]	0.083
aLVMI	0.90 [0.79, 1.01]	0.93 [0.81, 1.03]	0.89 [0.78, 1.02]	0.843	0.95 [0.81, 1.10]	0.98 [0.71, 1.18]	0.92 [0.77, 1.05]	0.232
LVH (*n*, %)	55/191 (28.8)	7/17 (41.2)	40/143 (28.0)	0.522	46/121 (38.0)	12/25 (48.0)	45/136 (33.1)	0.328

### Children with hypertension in the daytime period

We observed similar differences in age, and proportion of females, as for the 24-h period. Children with SH and IDH tended to have lower BMI *Z*-scores, while those with SDH had significantly lower BMI *Z*-scores compared to those with ISH (*P* < 0.05; Supplementary Table 2A). Regarding aLVMI, there were again no significant differences among ABP phenotypes in PH. Children with SH and IDH had the highest numerical aLVMi, but it was not statistically significantly different from ISH (mean difference = 0.025, 95% CI =  − 0.09 to + 0.15, *P* = 0.68). Children with SH and daytime IDH also had numerically the highest (although not statistically significantly different from ISH and SDH) proportion of LVH (Supplementary Table 2A). We again observed expected differences in absolute BP values and BP *Z*-scores across ABP groups for both PH and SH. These data are summarized in Supplementary Table S1B.

### Children with hypertension in the nighttime period

Children with PH and nighttime IDH were significantly younger, but there were no differences in the proportion of females with each ABP phenotype (Supplementary Table 2B). Children with SH and nighttime IDH, or SDH, had a lower BMI *Z*-score compared to ISH. aLVMi and the proportion of LVH were again not significantly different among ABP phenotypes. Differences in absolute BP and BP *Z*-score are summarized in Supplementary Table S1C.

### Overall prevalence of isolated ABP phenotypes in children with PH and SH

For those children with 24-h hypertension, we observed IDH in 34 (7%) of those with PH and 55 (12%) of those with SH (*P* < 0.01; Table 3). Further, of those with daytime hypertension and PH, 19 (8%) children had IDH, while 46 (12%) of those with SH had IDH (*P* = 0.08). Lastly, of those with nighttime hypertension, we observed that 19 (6%) had PH and IDH, while 63 (11%) had SH and IDH (*P* < 0.05). Further comparisons were made among those children with 24-h, daytime, and nighttime ISH or SDH, and these data are summarized in Table 3.

**Table 3:** Ambulatory blood pressure phenotype prevalence. ISH, isolated systolic hypertension; IDH, isolated diastolic hypertension; SDH, combined systolic and diastolic hypertension; %, total percentage of patients; PH, primary hypertension; SH, secondary hypertension. IDH values in bold. Comparison made between PH and SH. *P*, *P* value. *P* < 0.05*, *P* < 0.01**

	**24-h**	***N***	**%**	***P***	**Day**	***N***	**%**	***P***	**Night**	***N***	**%**	***P***
**ISH**	PH *N* = 524	291	56	**	PH *N* = 248	156	63	**	PH *N* = 321	163	51	*
	SH *N* = 466	213	46		SH *N* = 371	186	50		SH *N* = 563	245	44	
**IDH**	**PH** ***N* = 524**	**34**	**7**	**	**PH** ***N* = 248**	**19**	**8**	**0.08**	**PH** ***N* = 321**	**19**	**6**	*
	**SH** ***N* = 466**	**55**	**12**		**SH** ***N* = 371**	**46**	**12**		**SH** ***N* = 563**	**63**	**11**	
**SDH**	PH *N* = 524	199	38	ns	PH *N* = 248	73	29	*	PH *N* = 321	139	43	ns
	SH *N* = 466	198	43		SH *N* = 371	139	38		SH *N* = 563	255	45	

## Discussion

The key findings in our study include the observation of a varying prevalence of IDH across ABP phenotypes (6–12%). There was a lower prevalence of IDH in those with PH (6–8%), but a higher prevalence of IDH in children with SH (11–12%). Further, we observed differences in the prevalence of IDH because of biological sex, noting that females tended to have IDH more than males. Importantly, we also saw that the risk of LVH in our cohort was similar across all ABP phenotypes.

Our results agree with Rasała et al. [6], who observed higher DBP loads and levels in patients with SH using ABPM. We similarly observed a higher prevalence of ambulatory IDH in those with SH. Alsaeed et al. observed a similar low prevalence of IDH [5]; however, they used NHANES office data.

Knowing that differences in prevalence of hypertension based on sex have been described in the literature, we next sought to determine if there were sex-related prevalence differences between the ABP phenotypes, and specifically IDH, in our cohort. When further considering the observations of Alsaeed et al., [5] whereby they reported on an IDH phenotype using office BP measurements (more likely female, leaner, lower BMI), our study adds value in that we also observed this for those children with 24-h hypertension and both PH and SH. When comparing those with daytime hypertension and PH, there were more females with SDH; however, this was not the case for those with SH where children with IDH were again more likely to be female, like our observations for those with 24-h hypertension. There did not appear to be a sex predilection when comparing across ABP phenotypes and nighttime PH or SH. Intriguingly, the opposite was seen in Ali et al.’s study [14], and in a large study of Chinese adults, with more men having IDH [15].

It appears that not only is sex a possible factor but also age appears to be important. Alsaeed et al. [5] also observed that children with IDH tended to be younger. We did not observe that children with 24-h IDH, or daytime IDH were younger. However, those with nighttime IDH and either PH or SH were younger than those with ISH (*P* < 0.05).

Most importantly, a key finding from our study is that children with IDH on ABPM (either 24-h, daytime, or nighttime) had similar LVMI, aLVMI, and prevalence of LVH to those with ISH and SDH. This contrasts with the existing literature. The impact of increased blood pressure on HMOD in children with PH was assessed by Hamdani et al. [16], where they evaluated HMOD in participants who had ABPM and echocardiography within weeks of one another and concluded that DBP had no additive value in the prediction of LVH [16]. Other pediatric studies have looked at DBP and identified this to be non-consequential for LVH or HMOD [4, 17]. Specifically, Obrycki et al. studied ~ 300 pediatric patients with PH, assessing the impact of pre-hypertension on HMOD [4] and observed that 24-h DBP was not a predictor of negative changes in pulse wave velocity (PWV) or carotid intima-media thickness (cIMT), nor LVH [4]. Our results may differ owing to a larger sample size with a wider range of ages enrolled in this cohort.

While it requires further investigation, it would appear that IDH has an impact on LVH in both PH and SH and as such may have treatment implications. Drawing on literature from adult patients, it is clear that physicians have differing views on the importance of IDH with respect to HMOD and cardiovascular risk [18, 19]. One of the important takeaways from recent literature was the apparent increased risk for young patients with IDH. Indeed, in a study by Lee et al., those young adult patients (20–39 years old) whose BP category was Stage I diastolic hypertension had an increased estimated hazard ratio (HR) for cardiovascular disease (CVD) events and those with Stage II diastolic hypertension had an even higher risk [19, 20]. However, it is unclear whether these patients had PH or SH. Furthermore, when assessing the impact of BP change on risk of CVD events, Lee et al. were also able to show that by reducing DBP from Stage I hypertension to normotension, patients had a lower risk of CVD events compared to those that maintained Stage I [20]. Contrary to this, Jacobsen [21] argued in a recent adult cohort study and meta-analysis that isolated diastolic hypertension only appears to be important in those with controlled systolic hypertension and a DBP > 90 mmHg. He also argued that when using the AHA definition of IDH, there were no consistent, statistically significant associations with coronary artery calcification or incident cardiovascular events. However, they concluded that further studies in younger adults (and thus in children and adolescents) to better understand the risk of IDH were required.

The strengths of our study include the international, multicenter nature allowing for representation from multiple countries. Further, to our knowledge, this is one of the largest studies addressing ambulatory isolated diastolic hypertension with echocardiographic data in children. However, our study is not without limitations which are mainly the result of the retrospective design. We were unable to have our echocardiographic data validated by an independent examiner. Another limitation includes the inherent accuracy issues with oscillometric DBP measurements. Further, in some instances, the timing between ABPM and echocardiography was longer than 6 months (~ 10%); however, we reanalyzed the data using a strict 6-month cutoff between ABPM and echocardiography and the overall message was the same: the risk of LVH was no different between those with ISH, SDH, or IDH. Another limitation is the modification of guidelines around which patient should have an echocardiogram (across the 10-year enrollment period). We also did not assess for the impact of race, as this was not a variable reliably collected from all centers. Lastly, some children were treated for their hypertension, while others were not, or the data are not available. Future prospective studies are required to fully delineate the impact of IDH on LVH in children and adolescents.

In summary, we have analyzed data from a large, multi-center, international cohort of children, with ABPM and echocardiogram. To our knowledge, this is one of the largest cohorts of pediatric patients with nearly complete data allowing for direct comparison of ABP phenotypes, between those with PH and SH, to aLVMI. Our key findings were that ambulatory IDH was observed in ~ 10% of all patients in this cohort, there were more females with IDH, and children with ambulatory IDH, regardless of PH or SH, had essentially equal LVMI, aLVMI, and LVH risk. Although some studies have shown no strong association between DBP and LVH, our study shows that children with IDH have a similar prevalence of LVH when compared to ISH and SDH.

## Supplementary Information

Below is the link to the electronic supplementary material.

Graphical abstract (PPTX 227 KB)

Supplementary file 2 (DOCX 25 KB)

## Data Availability

The data are available upon reasonable request.
