# Supplementary material for: Ambulatory isolated diastolic hypertension and risk of left ventricular hypertrophy in children with primary and secondary hypertension
Source: Pediatr Nephrol. 2024 Jul 18;39(12):3533–41. doi: 10.1007/s00467-024-06457-8 (PMC11511691; doi:10.1007/s00467-024-06457-8)
Supplement: Supplementary file 1 — Graphical abstract (PPTX 227 KB) [file 467_2024_6457_MOESM1_ESM.pptx]

## Slide 1
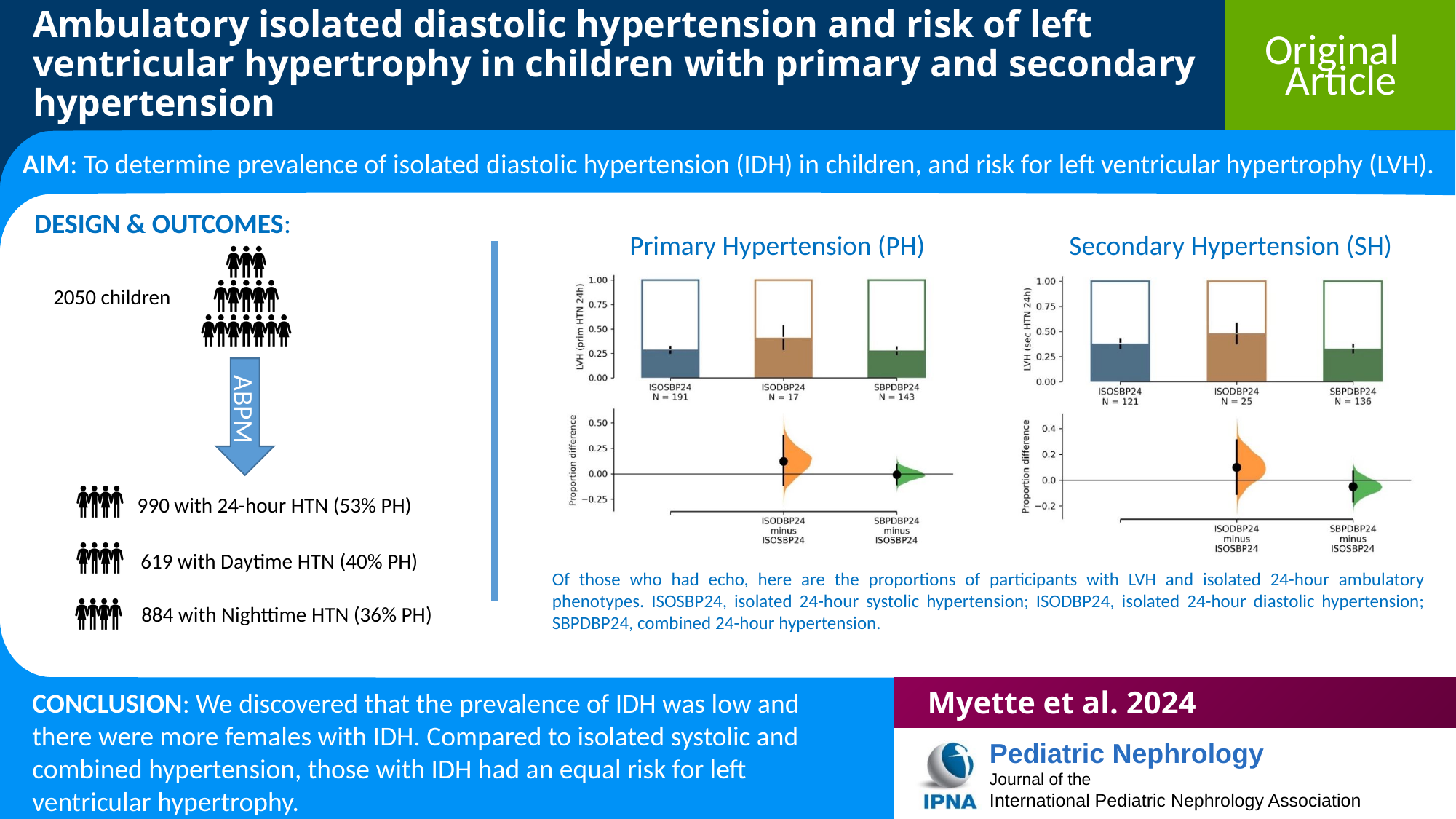

Ambulatory isolated diastolic hypertension and risk of left ventricular hypertrophy in children with primary and secondary hypertension
AIM: To determine prevalence of isolated diastolic hypertension (IDH) in children, and risk for left ventricular hypertrophy (LVH).
DESIGN & OUTCOMES:
Primary Hypertension (PH)
Secondary Hypertension (SH)
2050 children
ABPM
990 with 24-hour HTN (53% PH)
619 with Daytime HTN (40% PH)
Of those who had echo, here are the proportions of participants with LVH and isolated 24-hour ambulatory phenotypes. ISOSBP24, isolated 24-hour systolic hypertension; ISODBP24, isolated 24-hour diastolic hypertension; SBPDBP24, combined 24-hour hypertension.
884 with Nighttime HTN (36% PH)
Myette et al. 2024
CONCLUSION: We discovered that the prevalence of IDH was low and there were more females with IDH. Compared to isolated systolic and combined hypertension, those with IDH had an equal risk for left ventricular hypertrophy.
