# Supplementary material for: Ambulatory isolated diastolic hypertension and risk of left ventricular hypertrophy in children with primary and secondary hypertension
Source: Pediatr Nephrol. 2024 Jul 18;39(12):3533–41. doi: 10.1007/s00467-024-06457-8 (PMC11511691; doi:10.1007/s00467-024-06457-8)
Supplement: Supplementary file 2 — (DOCX 25 KB) [file 467_2024_6457_MOESM2_ESM.docx]

**Supplementary Table 1.** Absolute blood pressure and blood pressure Z-score data for the overall cohort stratified by ABPM classification. 24-hour (Table S1A); Daytime (Table S1B); Nighttime (Table S1C). Mean (SD); Median [IQR]; n (%). SBP, systolic blood pressure. DBP, diastolic blood pressure.

| **Table S1A** | **Primary** | | | **Secondary** | | |
| --- | --- | --- | --- | --- | --- | --- |
| **24-hour** | **24-h ISH** | **24-h IDH** | **24-h SDH** | **24-h ISH** | **24-h IDH** | **24-h SDH** |
| **N (%)** | 291 (55.5%) | 34 (6.5%) | 199 (38%) | 213 (45.7%) | 55 (11.8%) | 198 (42.5%) |
| **24-h SBP** | 131.00  [127.00, 135.00] | 122.00  [120.00, 123.75] | 135.00  [130.50, 139.00] | 128.00  [125.00, 133.00] | 119.00  [114.00, 123.00] | 133.00  [127.25, 140.00] |
| **24-h SBP Z-Score** | 1.93  [1.35, 2.41] | 1.15  [0.77, 1.51] | 2.38  [1.83, 3.28] | 2.02  [1.37, 2.54] | 1.08  [0.44, 1.49] | 2.68  [2.07, 3.40] |
| **24-h DBP** | 70.00  [67.00, 72.00] | 79.00  [76.00, 81.75] | 79.00  [76.00, 83.00] | 70.00  [66.00, 73.00] | 78.00  [76.00, 80.00] | 82.00  [78.00, 88.75] |
| **24-h DBP Z-Score** | 0.41  [-0.17, 0.85] | 2.09  [1.50, 2.46] | 1.97  [1.48, 2.62] | 0.51  [-0.29, 0.95] | 1.93  [1.67, 2.37] | 2.57  [1.81, 3.58] |

| **Table S1B** | **Primary** | | | **Secondary** | | |
| --- | --- | --- | --- | --- | --- | --- |
| **Daytime** | **Daytime ISH** | **Daytime IDH** | **Daytime SDH** | **Daytime ISH** | **Daytime IDH** | **Daytime SDH** |
| **N (%)** | 156 (62.9%) | 19 (7.7%) | 73 (29.4%) | 186 (50.1%) | 46 (12.4%) | 139 (37.5%) |
| **Daytime SBP** | 134.50  [131.00, 139.00] | 126.00  [122.50, 128.00] | 139.00  [135.00, 145.00] | 133.00  [131.00, 137.00] | 124.00  [122.00, 126.75] | 138.00  [133.00, 145.50] |
| **Daytime**  **SBP Z-Score** | 1.79  [1.19, 2.11] | 0.97  [0.38, 1.20] | 2.31  [1.80, 2.92] | 1.79  [1.20, 2.27] | 1.12  [0.52, 1.42] | 2.45  [1.96, 3.38] |
| **Daytime DBP** | 73.00  [70.00, 76.00] | 83.00  [82.00, 86.00] | 84.00  [82.00, 88.00] | 73.00  [69.00, 77.00] | 82.00  [81.00, 86.00] | 86.00  [83.00, 94.00] |
| **Daytime**  **DBP Z-Score** | 0.03  [-0.54, 0.49] | 1.97  [1.60, 2.34] | 2.08  [1.53, 2.82] | 0.11  [-0.68, 0.67] | 1.73  [1.46, 2.37] | 2.46  [1.73, 3.81] |

| **Table S1C** | **Primary** | | | **Secondary** | | |
| --- | --- | --- | --- | --- | --- | --- |
| **Nighttime** | **Nighttime ISH** | **Nighttime IDH** | **Nighttime SDH** | **Nighttime ISH** | **Nighttime IDH** | **Nighttime SDH** |
| **N (%)** | 163 (50.8%) | 19 (5.9%) | 139 (43.3%) | 245 (43.5%) | 63 (11.2%) | 255 (45.3%) |
| **Nighttime SBP** | 117.00  [113.00, 121.00] | 108.00  [106.50, 109.50] | 124.00  [118.00, 132.00] | 116.00  [113.00, 120.00] | 105.00  [102.00, 108.00] | 122.00  [117.00, 128.50] |
| **Nighttime**  **SBP Z-Score** | 1.56  [0.98, 2.16] | 1.00  [0.74, 1.33] | 2.38  [1.75, 3.14] | 1.54  [0.97, 2.05] | 0.64  [0.26, 1.12] | 2.41  [1.78, 3.00] |
| **Nighttime DBP** | 59.00  [55.00, 61.50] | 67.00  [66.00, 71.00] | 69.00  [67.00, 75.00] | 59.00  [55.00, 62.00] | 69.00  [66.00, 71.00] | 71.00  [68.00, 77.00] |
| **Nighttime**  **DBP Z-Score** | 0.38  [-0.29, 0.96] | 1.86  [1.69, 2.40] | 2.14  [1.80, 2.96] | 0.44  [-0.18, 1.03] | 2.15  [1.80, 2.61] | 2.47  [1.87, 3.44] |

**Supplementary Table 2.** Anthropometric, biochemical and blood pressure data for the overall cohort stratified by ABPM classification. Daytime (Supplementary Table 2A); Nighttime (Supplementary Table 2B). Mean (SD); Median [IQR]; n (%). BMI, body mass index. LVMI, left ventricular mass index. aLVMI, adjusted LVMI. LVH, left ventricular hypertrophy. Not all participants had echocardiogram, denominator for LVH (%) calculation included. P value column, ANOVA across three groups. * = Significantly different from ISH, post-hoc (P<0.05). ISH used as the primary comparator due to highest number of patients. IDH highlighted in grey.

| **Supplementary Table 2A** | **Primary** | | |  | **Secondary** | | |  |
| --- | --- | --- | --- | --- | --- | --- | --- | --- |
| **Daytime** | **Daytime ISH** | **Daytime IDH** | **Daytime SDH** | **P** | **Daytime ISH** | **Daytime IDH** | **Daytime SDH** | **P** |
| **N (%)** | 156 (62.9%) | 19 (7.7%) | 73 (29.4%) |  | 186 (50.1%) | 46 (12.4%) | 139 (37.5%) |  |
| **Age (years)** | 14.96  [12.03, 16.59] | 15.85  [12.91, 16.80] | 15.98 *  [14.43, 17.17] | 0.009 | 15.00  [12.03, 16.72] | 14.58  [12.71, 16.83] | 15.06  [12.08, 16.94] | 0.987 |
| **Female Sex, n (%)** | 39 (25.0) | 8 (42.1) | 35 (47.9) * | 0.002 | 68 (36.6) | **26 (56.5) *** | 60 (43.2) | 0.043 |
| **BMI (kg/m^2^)** | 24.11  [20.60, 30.54] | 23.33  [19.72, 29.58] | 24.96  [20.08, 28.46] | 0.737 | 21.47  [18.87, 26.00] | 21.29  [16.99, 25.43] | 19.93 *  [17.20, 23.22] | 0.017 |
| **BMI Z-Score** | 1.47  [0.33, 2.66] | 1.44  [0.60, 2.20] | 1.28  [-0.01, 2.10] | 0.196 | 1.04  [-0.08, 1.92] | 0.51 [-0.63, 1.56] | 0.30 *  [-0.64, 0.98] | 0.001 |
| **BMI > 95%ile (n, %)** | 55 (48.7) | 7 (43.8) | 21 (39.6) | 0.545 | 43 (29.5) | 11 (24.4) | 22 (18.0) | 0.095 |
| **LVMI** | 35.65  [32.62, 39.55] | 35.50  [33.25, 40.59] | 36.35  [32.23, 43.87] | 0.929 | 37.03  [32.80, 44.14] | 38.28  [29.77, 45.64] | 34.72 *  [29.36, 41.87] | 0.014 |
| **aLVMI** | 0.92  [0.78, 1.04] | 0.90  [0.76, 1.00] | 0.91  [0.80, 1.11] | 0.976 | 0.95  [0.82, 1.08] | 1.00  [0.74, 1.18] | 0.87 *  [0.75, 1.04] | 0.031 |
| **LVH (n, %)** | 22/66 (33.3) | 3/9 (33.3) | 15/44 (34.1) | 0.996 | 43/106 (40.6) | 14/29 (48.3) | 32/106 (30.2) | 0.118 |

| **Supplementary Table 2B** | **Primary** | | |  | **Secondary** | | |  |
| --- | --- | --- | --- | --- | --- | --- | --- | --- |
| **Nighttime** | **Nighttime ISH** | **Nighttime IDH** | **Nighttime SDH** | **p** | **Nighttime ISH** | **Nighttime IDH** | **Nighttime SDH** | **p** |
| **N (%)** | 163 (50.8%) | 19 (5.9%) | 139 (43.3%) |  | 245 (43.5%) | 63 (11.2%) | 255 (45.3%) |  |
| **Age (years)** | 14.92  [13.43, 16.48] | **12.31 ***  **[9.87, 16.46]** | 15.52  [13.45, 16.91] | 0.104 | 15.46  [13.78, 16.78] | **11.84 ***  **[9.67, 16.30]** | 15.25  [12.74, 16.78] | <0.001 |
| **Female Sex, n (%)** | 50 (30.7) | 8 (42.1) | 59 (42.4) | 0.092 | 84 (34.3) | 23 (36.5) | 112 (43.9) | 0.08 |
| **BMI (kg/m^2^)** | 23.98  [21.23, 30.42] | 23.33  [18.12, 29.58] | 24.32  [20.10, 29.58] | 0.542 | 22.86  [19.77, 26.82] | **19.71 ***  **[16.70, 23.76]** | 21.16  [17.50, 24.55] | <0.001 |
| **BMI Z-Score** | 1.48  [0.31, 2.66] | 1.36  [-0.52, 2.08] | 1.60  [-0.20, 2.52] | 0.613 | 1.10  [0.11, 1.98] | 0.85  [-0.23, 1.46] | 0.44 *  [-0.50, 1.71] | 0.005 |
| **BMI > 95%ile (n, %)** | 53 (46.5) | 7 (43.8) | 51 (50.0) | 0.827 | 63 (31.5) | 13 (22.8) | 57 (25.9) | 0.293 |
| **LVMI** | 35.84  [32.72, 42.27] | 33.44  [27.19, 37.43] | 35.32  [30.99, 41.25] | 0.224 | 36.49  [32.15, 40.97] | 36.92  [32.67, 45.89] | 36.46  [30.81, 43.37] | 0.661 |
| **aLVMI** | 0.91  [0.78, 1.04] | 0.84  [0.70, 0.94] | 0.89  [0.77, 1.01] | 0.471 | 0.90  [0.79, 1.05] | 0.96  [0.85, 1.11] | 0.92  [0.75, 1.05] | 0.513 |
| **LVH (n, %)** | 24/71 (33.8) | 2/10 (20.0) | 15/57 (26.3) | 0.513 | 43/140 (30.7) | 10/25 (40.0) | 61/177 (34.5) | 0.596 |
